# Cervicothoracic spinal cord and pontomedullary injury secondary to high-voltage electrocution: a case report

**DOI:** 10.1186/1752-1947-6-296

**Published:** 2012-09-13

**Authors:** Harpreet K Johl, Adel Olshansky, Said R Beydoun, Richard A Rison

**Affiliations:** 1 University of Southern California, Keck School of Medicine, Los Angeles County Medical Center, 1510 San Pablo Street, Suite 643, Los Angeles, CA, 90033, USA; 2 University of Southern California, Keck School of Medicine, Los Angeles County Medical Center, 1520 San Pablo Street, Suite 3000, Los Angeles, CA, 90033, USA; 3 Presbyterian Intercommunity Hospital, 12401 Washington Boulevard, Whittier, CA, 90602, USA

## Abstract

**Introduction:**

High-voltage electrical injuries are uncommonly reported and may predispose to both immediate and delayed neurologic complications.

**Case presentation:**

We report the case of a 43-year-old Caucasian man who experienced a high-voltage electrocution injury resulting in ischemic myelopathy and secondary paraparesis.

**Conclusion:**

High-voltage electrocution injuries are a serious problem with potential for both immediate and delayed neurologic sequelae. The existing literature regarding effective treatment of neurologic complications is limited. Long-term follow-up and multidisciplinary management of these patients is required.

## Introduction

High-voltage electrical injuries are uncommonly reported and may lead to serious neurologic sequelae. The true incidence and prevalence of these events are difficult to ascertain. This may be due to the fact that these cases are underreported in the literature, probably due to population unawareness and medically underserved communities. Children and young men are at the highest risk to receive an accidental electrical injury, with these subgroups being less likely to report the incident [1]. The extent of injury may be apparent immediately or it may take weeks to manifest. Clinicians need to be aware of the neurological consequences of electrocution.

## Case presentation

A 43-year-old right-handed Caucasian man experienced an electrical burn after contact with a 440 volt line while working on a roof. Upon standing up from the squatted position, the right side of our patient’s head came into contact with an exposed wire, resulting in electrocution with a loss of consciousness. Witnesses called the emergency medical services, and our patient was intubated at the scene. He was transferred from a local community hospital to our institution’s burn unit for a higher level of care. Second and third degree burns covered 21% of his total body surface area. The entry wound was through his scalp, traversing through his neck and chest, into his groin, and exiting from his left foot. No spinal fractures were identified.

Initially, our patient was alert and able to move all four limbs. Over the ensuing days, he was observed to move his legs less than his arms. Two days after our patient received multiple skin grafts and a calvarial flap, sedation was held and he was noted to have absent limb movements during routine dressing changes. A neurological consultation was obtained on his 10th day of hospitalization because of his evolving weakness. On examination, our patient was intubated, and his head, chest, groin and bilateral lower extremities were covered by dressings. He was awake with eyes open, alert and able to follow simple commands. A cranial nerve examination revealed reactive pupils, intact extraocular movements and symmetrical facial movement to smile, eye closure and eyebrow elevation. Our patient indicated normal light touch sensation on his face with nodding. He was breathing over the ventilator and able to shrug his shoulders. A motor examination showed normal muscle bulk with flaccid tone and only intermittent, trace movement in his left fingers. Our patient acknowledged pain with nodding on light pressure to all limbs. His deep tendon reflexes were diminished and plantar responses were equivocal.

**Figure 1:**
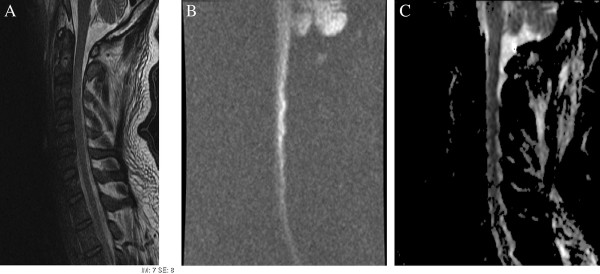
**Magnetic resonance imaging of the cervical spine repeated eight days after the initial magnetic resonance image performed on admission.** Interval development of cord edema and infarction is seen from the C2 to T2 levels. (**A**) Sagittal T2 sequence. (**B**) Diffusion-weighted image. (**C**) Apparent diffusion coefficient image. The diffusion-weighted image and apparent diffusion coefficient image do not demonstrate the exact same level of involvement, with the apparent diffusion coefficient image showing signal changes slightly lower in the cervical spine than the diffusion-weighted image. Also, the diffusion-weighted image signal abnormality does affect somewhat the dorsal aspect of the cord. This reflects the often diffuse and collateral nature of electrical injuries.

**Figure 2:**
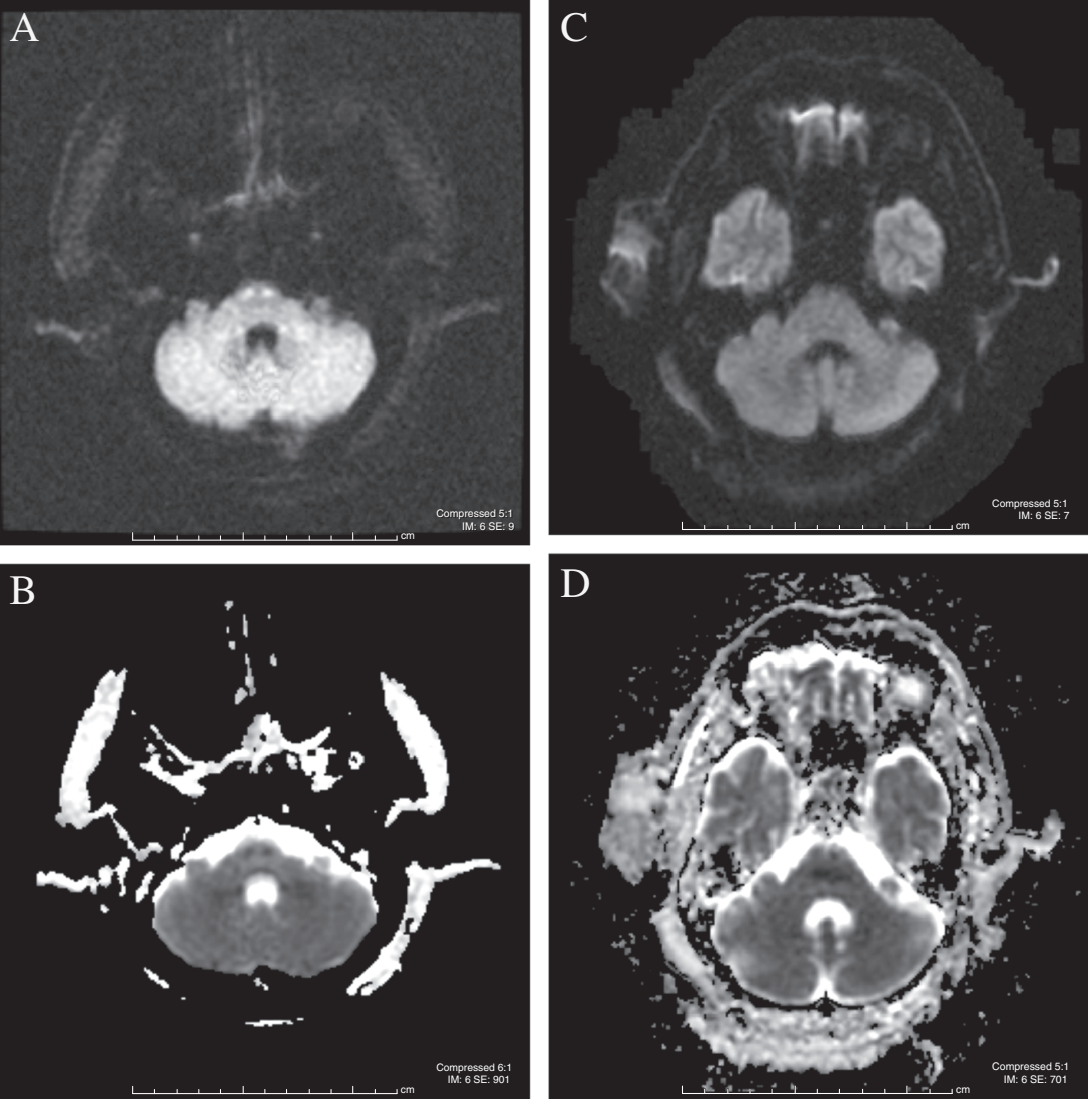
**Magnetic resonance imaging of the brain demonstrating restricted diffusion in the bilateral medullary pyramids and pons.** (**A**) Diffusion-weighted image. (**B**) Apparent diffusion coefficient image. Repeat magnetic resonance imaging of the brain performed two months later demonstrates resolution of the diffusion restriction: (**C**) diffusion-weighted image; (**D**) apparent diffusion coefficient image.

On admission, magnetic resonance imaging (MRI) of his cervical spine demonstrated changes suggestive of edema from C3 to T2 levels with an intact spinal cord. Given the changes noted on the neurological examination, a repeat MRI was suggested. Repeat imaging performed eight days after the first MRI revealed an interval development of cord swelling and acute ischemic infarction (Figure 1 A,B,C). MRI of his brain showed restricted diffusion in the bilateral medullary pyramids and pons, indicating infarction of the corticospinal tracts at the pontomedullary level (Figure 2 A,B).

**Figure 3:**
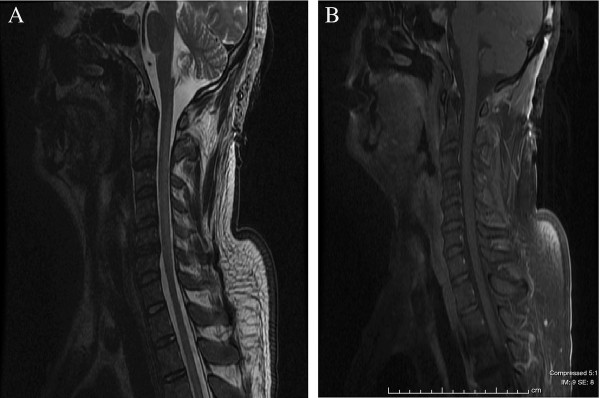
**Magnetic resonance imaging of the cervical spine two months after the image in Figure**1**was taken.** (**A**) T2 sequence. (**B**) T1 post-contrast image.

Our patient was treated with fluid resuscitation, electrolyte replacement and nutritional support as well as wound care and pain control. Intravenous steroids were considered however not pursued due to the risk of infection and imaging findings of ischemia. A tracheostomy and percutaneous gastrostomy tube were placed. Our patient’s hospital course was complicated by pneumonia and the development of pulmonary emboli, requiring placement of an inferior vena cava filter. Over the next two months, our patient showed minimal improvement in motor function. Repeat imaging was obtained. MRI of his brain revealed near complete resolution of the ischemic changes (Figure 2 C,D). A cervical spine MRI suggested a persistent, abnormal signal posteriorly from the cervicomedullary junction to the C6 level with an interval decrease in cord edema (Figure 3 A,B). His course showed an ability to hold his head up with more movement in his left arm against gravity; some flexion of his right elbow and wrist was also noted. No improvement was observed in his lower extremities. Our patient was subsequently transferred to an inpatient rehabilitation facility, and unfortunately was lost to follow-up thereafter.

## Discussion

In electrocution injuries, the overall mortality may reach 5%. Electrical injuries have been reported to occur in low-voltage settings, such as with household use, and high-voltage exposures from occupational hazards and lightning strikes [1]. Given the nature of these injuries, most of the literature has been reported as case studies with limited data regarding the evaluation and treatment of neurological complications.

Several pathophysiological mechanisms of injury to the nervous system have been proposed, including thermal injury, electroporation, and vascular damage through direct injury as well as indirect injury.

Electrical current flows from an area of low resistance to high resistance. Low areas of resistance include muscle, nerve and blood vessels. High-resistance tissue includes skin, connective tissue and bone, which suffer greater heat injury, typically at entry and exit sites but damage may be caused in any structure along the path of the current [2-4]. Nervous tissue provides a low-resistance route for electrical current. As the electrical current travels through tissue, neurons with larger surface area are more likely to be damaged by electroporation, in which the increase in cellular permeability and conductivity caused by permanent conformational change to membrane proteins ultimately leads to cell death [5]. The heat loss that occurs as current flows through tissues of increasing resistance causes damage to the intima and adventitia of the vasculature, such as thrombosis, necrosis of the vascular wall, vasospasm and spread into nearby tissue [3,4]. Most patients survive the initial insult, limiting the pathological work-up, with few postmortem studies available for review [1]. Morbidity of electrocution has been related to electrothermal injury causing direct tissue damage with secondary ischemic changes from vascular insult. An accurate prognosis is challenging given the variations in the duration and extent of injury, the frequency of current, and the anatomic site. There are relatively few reports that correlate the clinical, electrophysiological and imaging changes that occur with electrocution injuries [6].

Classification of injuries has been divided by onset of symptoms. Silversides [7] divided the stages into immediate, secondary and late effects. Immediately after an electrical current passes through the human body, thermal injury occurs within nerve cells, manifesting effects such as altered sensorium and/or loss of consciousness, severe pain, hearing and vision changes, motor signs (including paralysis), respiratory compromise, or sensory complaints. Recovery occurs within 24 hours. Secondary effects include temporary paralysis and autonomic disturbances. The late effects are noted to start after five days, manifesting as hemiplegia, movement disorders, brainstem dysfunction and cranial neuropathies [4,7].

Spinal cord injuries have also been classified into transient and permanent disability. Motor deficits occur more often than sensory disturbances [8] and are secondary to vascular damage incurred by the anterior spinal artery and its branches [4,8-12], which supply two thirds of the spinal cord, including the lateral corticospinal tracts, spinothalamic tracts and the anterior horn cells along with the central gray matter of the spinal cord. This hypothesis may be supported by the susceptibility of smaller lumen vessels to injury, with less dissipation of heat to surrounding structures compared with their larger vessel counterparts [7].

Ko et al. carried out a retrospective study of spinal cord injuries related to electrical burns. They reported that 11 out of 13 patients with entry wounds in the head and neck region were found to have quadriplegia with exit sites located in the upper extremities, and paraplegia with exit sites located in the lower extremities. Most of these patients were noted to have hypotonia acutely in the first two to ten days after injury in an ascending pattern. It was postulated that this pattern of injury was related to ischemic damage to the arterial blood supply and vulnerability in the spinal cord. These findings provided the rationale to administer prostaglandin E1 (10μg/day for three weeks) or steroid therapy to reduce ischemic injury to the spinal cord [4].

Early discovery of extensive myelopathy of the cervical spine is aided by neuroimaging. Most of the described cases used T2 hyperintensities, correlating to clinical deficits [5]. Initial imaging of our patient did not show any significant changes to explain his quadriparesis. However, repeat imaging performed roughly one week after initial MRI of his brain and cervical spine showed extensive involvement of the pyramidal tracts as well as cord edema, which explained our patient’s clinical findings. It was felt that the bilateral lesions involving the corticospinal tracts particularly contributed to our patient’s paresis. As with our case, serial imaging may prove essential for identifying delayed sequelae associated with electrocution injuries. A differential diagnosis made with radiology may be wide and includes neoplasm, infarction and electrocution injury. Repeat imaging may assist in making the correct diagnosis. Exploring additional neuroimaging modalities may be beneficial, such as magnetic resonance spectroscopy. Electrodiagnostic studies, such as evoked potentials, nerve conduction studies and electromyography may have prognostic value. The latter two modalities can help determine the severity of motor axonal loss, stemming from anterograde anterior horn cell degeneration in the ventral gray matter. Technical aspects limit the value of these studies secondary to the presence of skin injury and accessibility.

No established guidelines are available in the literature regarding the treatment of high-voltage electrical injury. Each case has been treated with supportive care. Early treatment of electrical injury starts with initial fluid resuscitation, respiratory support and prevention of infection. The lack of systematic guidelines makes clinical care for the patient with electrocution injuries challenging. Work-up of the neurological deficit with imaging or electrodiagnostic studies, treatment, and prognosis for recovery is dictated by the personal experience of the physician. There are no randomized double-blinded trials available on electrocution. The majority of the information comes from isolated cases, case series and animal experimental models, with fewer articles available in the radiology and neurological literature.

No consensus exists regarding medication use. High-dose intravenous steroids and prostaglandin E1 were cited in the literature as early treatment modalities. Neurological morbidity has been variable. Some case studies with delayed findings of severe myelopathy due to electrocution injury did not show full recovery [4] while other case reports showed near complete resolution of motor deficits with aggressive physical therapy [13]. Multidisciplinary management approaches involving the intensivist, neurologist, plastic surgeon, physical and occupational therapists, and psychologist, as well as a nutritionist, is necessary to achieve the best possible outcomes. Long-term neurological follow-up is justified by the rich body of evidence of delayed complications cited in the literature.

## Conclusion

High-voltage electrocution injuries are a serious problem with potential for both immediate and delayed neurologic sequelae. The existing literature regarding effective treatment is limited. Long-term follow-up and multidisciplinary management of these patients is needed.

## Consent

Written informed consent was obtained from the patient for publication of this case report and accompanying images. A copy of the written consent is available for review by the Editor-in-Chief of this journal.

## Competing interests

HJ, AO, and SRB declare that they have no competing interests. RAR is a Deputy Editor for the *Journal of Medical Case Reports* and is an Associate Neurology Editor for *Case Reports in Neurology* and *Grand Rounds*.

## Authors’ contributions

HKJ and AO examined the patient and wrote the initial manuscript. SRB supervised the coordination of clinical care and reviewed the manuscript. RAR rewrote and edited the manuscript, performed an additional literature search, and examined and interpreted the magnetic resonance imaging scans. SRB and RAR were responsible for the intellectual content of the paper along with critical appraisals, suggestions, and revisions. All authors significantly contributed to, read and approved the final version of the manuscript.
